# miR-20a Overexpression in Adipose-Derived Mesenchymal Stem Cells Promotes Therapeutic Efficacy in Murine Lupus Nephritis by Regulating Autophagy

**DOI:** 10.1155/2021/3746335

**Published:** 2021-10-21

**Authors:** Shanshan Wei, Zhiwen Zhang, Lu Yan, Yinjuan Mo, Xianwen Qiu, Xiangbin Mi, Kuan Lai

**Affiliations:** ^1^Department of Dermatology, Zhujiang Hospital, Southern Medical University, China; ^2^Department of Dermatology, Nanfang Hospital, Southern Medical University, China

## Abstract

**Objective:**

Lupus nephritis is the most common and severe complication of systemic lupus erythematosus. The aim of our study was to investigate the efficacy of miR-20a overexpressing adipose-derived stem cell (ADSC) transplantation in murine lupus nephritis (LN) and explore potential molecular mechanisms.

**Methods:**

Mouse ADSCs were transfected with a miR-20a lentiviral vector to obtain miR-20a overexpression ADSCs (miR-20a-ADSCs). We first observed the influence of miR-20a on ADSC viability and apoptosis *in vitro*. B6.MRL/lpr mice were administered ADSC/miR-20a-ADSC intravenously every week from age 30 to 33 weeks, and the lupus and normal control groups received PBS on the same schedule.

**Results:**

miR-20a expression increased in miR-20a-ADSC-derived exosomes, and miR-20a overexpression promoted ADSC proliferation and inhibited apoptosis. Compared with ADSCs, miR-20a-ADSC treatment significantly improved serologic and histologic abnormalities, as evidenced by reduced serum creatinine, anti-dsDNA antibody, 24 h urine protein levels, nephritis scores, and C3/IgG deposits. Furthermore, miR-20a-ADSC treatment resulted in downregulated Akt, mTOR, and p62 expression and upregulated miR-20a, Beclin 1, and LC3 II/I expression compared with ADSC treatment. After treatment with miR-20a-ADSC, a significant increase in the number of autophagosomes within podocytes was observed, along with upregulated expression of podocin and nephrin, compared with the ADSC group.

**Conclusions:**

miR-20a-ADSC transplantation prevents the development of lupus nephritis and significantly ameliorates already-established disease, and its mechanism is related to autophagy by targeting the miR-20a-regulated mTOR pathway.

## 1. Introduction

Systemic lupus erythematosus (SLE) is a clinically heterogeneous autoimmune disease that affects multiple organs and systems, has limited treatment options, and has no cure. The standard therapy for LN patients includes immunosuppressive regimens and corticosteroids. However, despite an aggressive regimen, about 20% of LN patients do not respond to standard therapy [1]. Within 10 years of an initial SLE diagnosis, 5%–20% of patients with LN develop end-stage kidney disease, and multiple comorbidities associated with immunosuppressive treatment, including infections, osteoporosis, and cardiovascular and reproductive effects, remain a concern [2]. Considering these factors, more effective therapies are needed.

Recent years have witnessed an increase in interest in developing stem cell transplantation for LN [3]. Mesenchymal stem cells (MSCs) are of clinical interest due to their multilineage differentiation, immune hyporesponsiveness, and multipotency, derived from murine bone marrow, adipose tissues, and human embryos [4, 5]. Current evidence demonstrates that MSCs release a type of specialized extracellular vesicle known as exosomes to provide therapeutic benefits. Exosomes are membranous nanosized vesicles secreted by a variety of cells, merging their membrane contents into the recipient cell membrane and transferring factors into recipient cells. These have been extensively reported as the principal therapeutic agent mediating MSC paracrine action, which underpins the therapeutic capabilities of MSCs in inhibiting apoptosis, reducing injury, or promoting repair in recipient cells [6]. Adipose tissue seems to be the most advantageous tissue from which to isolate them because of its abundance, its subcutaneous location, and the need for less invasive techniques. Adipose tissue-derived stem cells (ADSCs) are therefore considered to be of clinical use in regenerative medicine [7, 8]. In our previous study, we found that ADSC (passage 6) transplantation decreases IL-17 expression in MRL/lpr mice [9] and ADSC (passage 4) transplantation prevents LN development and ameliorates already-established disease via the mTOR/HIF-1*α* signal pathway [10]. However, there is a certain recurrence rate, and only a small percentage of cases are clinically cured without oral medication after MSC transplantation. To enhance their therapeutic efficacy, interventions through endogenous genetic modification and exogenous treatment are warranted.

miRNAs are essential modulators of many genes and biological processes and can influence cell apoptosis, proliferation, and immunomodulatory properties [11]. Recent studies provide evidence that miRNAs can enhance the effectiveness of MSCs. miR-20a, a member of the miR-17-92a cluster, significantly enhances the survival of MSCs in intracerebral hemorrhage, and miR-20a-overexpressing MSCs clearly improved neurological function in intracerebral hemorrhage rats [12]. Furthermore, miR-20a expression is significantly downregulated in SLE patients compared with healthy controls, indicating that the miR-20a agomir mitigates lupus [13, 14]. Nevertheless, the role of miR-20a in ADSCs is unknown. In this study, we investigated the therapeutic effect of miR-20a-overexpressing ADSCs (miR-20a-ADSC) in murine LN and explored its underlying mechanism.

The activated mTOR pathway has been detected in LN and has emerged as a central pathway for pathogenesis and treatment with rapamycin or other mTOR inhibitors, displaying protective and beneficial effects for LN [15, 16]. In our previous study, ADSC (passage 4) transplantation prevented LN development and ameliorated already-established disease via the mTOR/HIF-1*α* signal pathway [10]. In addition, mTOR is a transcriptional regulator of autophagy [17, 18], and more recently, genetic and cellular studies have suggested that defects in autophagy contribute to the pathogenesis of SLE, particularly in terms of adaptive immune response [19, 20]. In the present study, we aimed to investigate the therapeutic effect of miR-20a-ADSC treatment in LN. We upregulated miR-20a to demonstrate its role in ADSC viability and immunomodulatory property and explored the therapeutic mechanism in murine LN. This study offers a novel therapeutic method to enhance the efficacy of ADSCs in SLE.

## 2. Materials and Methods

### 2.1. Mouse ADSC Preparation

Mouse ADSCs were obtained from the axillary and inguinal subcutaneous fat of C57BL/6 mice at 3–4 weeks of age, as previously described [10]. Blood clots, unnecessary tissues, connective tissues, vessels, and skin were removed from the mouse adipose tissues. The tissues were washed thrice with phosphate-buffered solution (PBS) supplemented with antibiotics (penicillin/streptomycin) (100 IU/mL), homogenized, and then treated with 0.075% collagenase type I (Sigma-Aldrich, St. Louis, Missouri, USA) in PBS for 30 min at 37°C with gentle shaking. Collagenase was inactivated using an equal volume of Dulbecco's modified Eagle's medium containing high glucose (DMEM-HG, Gibco, USA) and 10% (*v*/*v*) fetal bovine serum (FBS, Gibco). The digested tissue was then centrifuged at 470g for 5 min. The cell pellet was resuspended in DMEM-HG with 10% (*v*/*v*) FBS and passed through a 100 mM filter to remove debris. The filtrate was again centrifuged at 470g for 5 min. The isolated stromal cells were resuspended in DMEM-HG containing 10% (*v*/*v*) FBS and seeded at a density of 4 × 10^4^ in 25 cm^2^ culture flasks. Experimental procedures were conducted in accordance with the regulations of the animal protection laws of China and approved by the animal ethics committee of Nanfang Hospital (NFYY-2019-105).

### 2.2. Lenti-miR-20a-Luciferase Construction and Cell Transfection

We used the pCDH-CMV-MCS-EF1-copGFP lentiviral vector (System Biosciences, Palo Alto, CA, USA) as the backbone plasmid. The miR-20a precursor sequence and vector were digested with *EcoR*I and *BamH*I and then ligated using T4 ligase (Takara, Kyoto, Japan). The pre-miR-20a amplification primers were as follows: sense 5′-AGAATTCTGTCGATGT AGAATCTGCCTG-3′ and antisense 5′-AGGATCCGACAGTTTGATTGGGCGACAG-3′. Sanger sequencing was used to verify the plasmid reconstruction. For lentiviral production, 293T cells were transfected with the lentiviral plasmid carrying pre-miR-20a fragments or control vector, along with the lentiviral packaging mix. The medium was changed 18 h after transfection, the cells were cultured for two more days, and the supernatant containing the viruses was harvested and concentrated. The lentivirus titer was titrated using a titer kit, and the titer was determined to be 1 × 10^9^/mL.

ADSCs were transfected with the lentiviral vector at passage 3. The lentiviral vectors carrying the pre-miR-20a fragments were added into the ADSCs at a multiplicity of infection (MOI) of 30.

After transfection for 24–48 h, green fluorescent protein expression was observed under a fluorescence microscope. The transfection efficiency was calculated. Transfection with the empty lentiviral vector served as the negative control (NC). miR-20a levels in each group were determined by quantitative reverse-transcription PCR (RT-PCR).

### 2.3. Cell Counting Kit-8 (CCK-8) Assay

The cell proliferation of different groups was assessed using CCK-8 (Dojindo, Shanghai, China) according to the manufacturer's instructions. A suspension of ADSCs was prepared, and 1 × 10^4^ cells per well were seeded into 96-well plates. The reaction was terminated, and the medium was discarded after 24 h of culture. Approximately 10 *μ*L of the CCK-8 solution was added to each well and incubated for 4 h. We measured the optical density (OD) at a wavelength of 450 nm.

### 2.4. Exosome Isolation

Exosomes from culture supernatants of ADSCs (passage 5) from different groups were isolated and cultured with 10% exosome-free FBS for 48 h. Exosomes were isolated from supernatants using an ExoQuick-TC isolation kit (System Bioscience, USA). We centrifuged the culture supernatants at 3,000g (15 min) to remove cell debris and then mixed the cell pellet with the total exosome isolation reagent overnight at 4°C. After centrifugation at 1,500g for 30 min, the supernatants were discarded, and the blank pipes were centrifuged at 1,500g for 5 min. Then, we isolated miRNA from exosomes using a mirVana miRNA isolation kit (Invitrogen, Carlsbad, CA, USA). miR-20a expression levels were analyzed by RT-PCR, and CD63 expression in exosomes was assessed by western blotting.

### 2.5. Mice, Experimental Groups, and Treatment Protocol

We purchased 22-week-old female B6.MRL/lpr mice and C57BL/6 mice from the Model Animal Research Center of Nanjing University (Nanjing, China) and the Laboratory Animal Research Center of Southern Medical University (Guangzhou, China), respectively.

The mice were randomized into four groups as follows: the lupus group (*n* = 10), ADSC group (treated with ADSC; *n* = 10), miR-20a group (treated with miR-20a-ADSC; *n* = 10), and normal control group (C57BL/6 mice; *n* = 10). From age 30 to 33 weeks, the ADSC/miR-20a group received an intravenous injection of 2 × 10^5^/10 g ADSCs/miR-20a-ADSC (passage5)/150 *μ*L PBS weekly. In the meantime, the normal control and lupus groups received intravenous injections of 150 *μ*L PBS weekly. The mice were sacrificed 14 days after the last intravenous injection.

### 2.6. Determination of 24 h Proteinuria

In our study, 24 h urine was collected from different groups weekly, metabolic cages were used beginning at 28 weeks, and the Coomassie brilliant blue method was used to measure urinary protein.

### 2.7. Determination of Anti-dsDNA Antibodies and Serum Creatinine

Blood samples were centrifuged, and the supernatant was collected. The levels of serum creatinine were evaluated using creatinine and urea commercial kits (Nanjing Jiancheng Bioengineering Institute, Nanjing, China) according to the manufacturer's instruction. Anti-dsDNA antibody concentrations were measured using a mouse anti-dsDNA ELISA kit (Cusabio, Wuhan, China).

### 2.8. Histological Evaluation of LN

Kidneys were embedded in paraffin and subjected to hematoxylin/eosin (HE) staining and immunohistochemistry. A blinded veterinary histopathologist analyzed and scored the HE-stained kidney sections at 400x magnification and from 20 high-power fields in each group. The severity of LN was evaluated on a scale of 0-6 scores as previously described [10].

### 2.9. Immunohistochemistry

Kidney sections were cut into 5 *μ*m thick slices. The sections were incubated with primary, rabbit anti-mouse C3 (Abcam, Cambridge, UK), or rabbit anti-mouse IgG (Abcam, Cambridge, UK) antibodies overnight at 4°C and subsequently incubated with HRP-conjugated secondary antibodies. Then, the samples were incubated with the 3,3-diaminobenzidine (DAB) peroxidase substrate kit (Boster, Wuhan, China). The area and integrated option density (IOD) were counted at 400x magnification from 20 high-power fields in each group using an Image-Pro Plus 6.0 photogram analysis system (IPP 6.0, Media Cybernetics, USA).

### 2.10. RT-PCR

Total RNA was isolated from kidneys using the TRIzol reagent (Life Technologies, Carlsbad, CA, USA). One microgram of total RNA was reverse transcribed (Takara, Otsu, Japan) and amplified with SYBR Premix Ex Taq™ (Takara) according to the manufacturer's instructions. The primers used in this study are shown in Table 1. Measurements were performed using the LightCycler 480 (Roche, Switzerland). Each sample was analyzed in triplicate. Products were normalized according to GAPDH expression, and analysis was performed using the 2^−ΔΔCt^ method.

### 2.11. Western Blot

Kidney tissues were homogenized in RIPA buffer (containing protease inhibitors) and stored at -80°C until analysis. Primary antibodies against GAPDH, cleaved caspase-3, Akt, p-Akt (Thr308), mTOR, p-mTOR (2448), Beclin 1, LC3, nephrin, and podocin (anti-NPHS2) (Abcam, Cambridge, UK) and SQSTM1/p62 (Cell Signaling, Danvers, MA, USA) were used. The detailed description of western blot analysis was performed as previously reported [10].

### 2.12. Transmission Electron Microscopy

For electron microscopy, kidney tissues were fixed in 3% glutaraldehyde and 1% osmium tetroxide. They were then dehydrated across an ethanol gradient and then washed with acetone. The samples were cut into 1 *μ*m ultrathin sections after embedding in Epon-812 and dyed with uranium acetate and plumbum citrate. The samples were observed using a transmission JEOL JEM-1400 Plus electron microscope; each kidney tissue sample was examined, and a copper mesh was randomly selected for counting the number of autophagosomes in podocytes (including foot processes).

### 2.13. Statistical Analyses

The *χ*^2^ test and Student's *t*-test were used to analyze the differences among groups. The data were expressed as the mean ± standard deviation. ANOVA, followed by the Bonferroni or SNK test, was performed for multiple comparisons. All data were analyzed using SPSS 21.0 software (IBM, SPSS, Chicago, IL, USA), and graphs were produced by GraphPad Prism 5.0 (GraphPad Software, Inc., La Jolla, CA, USA). *P* < 0.05 was considered statistically significant.

## 3. Results

miR-20a overexpression increases ADSC proliferation and inhibits apoptosis *in vitro*; miR-20a expression levels increased in miR-20a-ADSC-derived exosomes.

**Figure 1:**
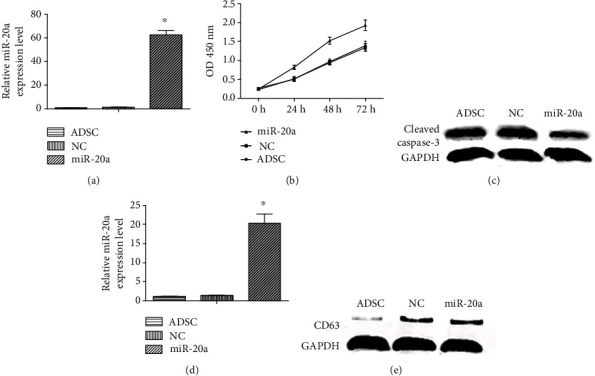
miR-20a overexpression promotes cell proliferation and inhibits apoptosis of ADSCs, and miR-20a-ADSC-derived exosomes exhibit upregulated miR-20a expression: (a) miR-20a expression levels; (b) cell proliferation; (c) cleaved caspase-3 expression levels in different groups; (d) miR-20a expression in exosomes; (e) CD63 expression in exosomes of the NC and miR-20a groups (the ADSC group consists of ADSCs only). The data are expressed as the mean ± SD. ^∗^*P* < 0.05*vs.* ADSC group. ADSC: adipose-derived stem cell group; miR-20a: miR-20a-ADSC group; NC: negative control group.

To investigate the therapeutic effect of miR-20a-ADSC, ADSCs were transfected with lentivirus-miR-20a and empty lentivirus vectors. Transfection efficiency was evaluated by RT-PCR, which showed that miR-20a-ADSC elevated the expression of miR-20a by at least 62.5-fold relative to the ADSC and NC groups (*P* < 0.05; Figure 1(a)).

We also assessed the effect of miR-20a on cell proliferation using CCK-8. We observed that cell proliferation was not significantly different between the ADSC and NC groups (*P* > 0.05; Figure 1(b)) and significantly increased after miR-20a overexpression. Furthermore, we found that cleaved caspase-3, an important protein involved in the process of apoptosis, was downregulated in the miR-20a group compared with the NC group (Figure 1(c)).

To investigate whether the role of miR-20a was mediated by exosomes, we isolated the exosomes in different groups. The exosome marker CD63 was used to verify exosomes (Figure 1(e)). We measured miR-20a expression levels in exosomes derived from different groups. Our results showed that the expression of miR-20a significantly increased in miR-20a-ADSC-derived exosomes compared with the NC and ADSC groups (*P* > 0.05; Figure 1(d)).

### 3.1. miR-20a-ADSC Treatment Reduces Disease Severity and Delays LN Disease Progression in Murine LN

**Figure 2:**
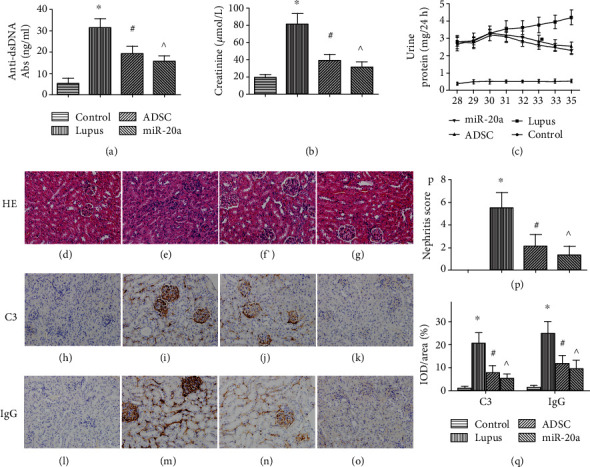
miR-20a-ADSC/ADSC treatment delays disease progression and reduces disease severity in murine LN: (a) anti-dsDNA, (b) serum creatinine, and (c) 24 h urine protein. (d–o) Representative images of kidney sections stained with H&E and immunohistochemistry of the normal control group (d, h, and l), lupus group (e, i, and m), ADSC group (f, j, and n), and miR-20a group (g, k, and o). (p) Nephritis severity scores. (q) Measurement of C3- or IgG-positive cells using IOD/area. The data are expressed as the mean ± SD. ^∗^*P* < 0.05*vs.* control group; ^#^*P* < 0.05*vs.* lupus group; ^*P* < 0.05*vs.* ADSC group. Control: normal control group; lupus: lupus group; ADSC: adipose-derived stem cell group; miR-20a: miR-20a-ADSC group.

We measured the anti-dsDNA antibody and creatinine levels to evaluate autoantibody levels and kidney function. The level of anti-dsDNA was significantly lower in the ADSC (19.17 ± 3.59 ng/mL) and miR-20a (15.57 ± 2.62 ng/mL) group compared with the lupus group (31.39 ± 4.2 ng/mL) and was the lowest in the miR-20a group (all *P* < 0.05; Figure 2(a)). The anti-dsDNA level was normal in the normal control group.

The serum creatinine level was 19.15 ± 3.10 *μ*mol/L in the normal control group, which was significantly lower than those in the lupus (80.98 ± 12.48 *μ*mol/L), ADSC (39.15 ± 6.76 *μ*mol/L), and miR-20a (31.20 ± 6.01 *μ*mol/L) groups. Serum creatinine levels decreased in the ADSC and miR-20a groups compared to the lupus group and were the lowest in the miR-20a group (all *P* < 0.05; Figure 2(b)).

We measured 24 h urine protein in different groups from 28 to 35 weeks (Figure 2(c)). Prior to ADSC treatment, the 24 h urine protein levels were similar in the miR-20a, ADSC, and lupus groups. Then, it decreased after the first administration (31 weeks) and was significantly lower after the second administration (32 weeks) in the ADSC group compared to the lupus group, and the miR-20a group had the lowest level among the three groups (Figure 2(c)). The 24 h urine protein was normal in the normal control group.

### 3.2. Effect of miR-20a-ADSC on Renal Histopathology and Immunopathology

In the lupus group, we found glomerular mesangial cell and mesangial matrix proliferation, inflammatory cell infiltration, and necrosis using HE staining, while there were fewer histopathologic abnormalities in the ADSC/miR-20a-ADSC group (Figures 2(d)–2(g)). The nephritis score was 5.50 ± 1.38 in the lupus group, which was significantly higher than those in the ADSC (2.11 ± 1.02) and miR-20a (1.33 ± 0.77) groups (all *P* < 0.05; Figure 2(p)). The nephritis scores were lower in the miR-20a group compared with the ADSC group, and no renal pathology was observed in the normal control group (Figure 2(p)).

C3 and IgG deposits were mainly observed in the glomeruli, along with a small amount in the tubular lesions, which were analyzed by the IOD/area (Figures 2(h)–2(o)). The C3 deposits in the glomeruli were 1.02 ± 0.74 in the normal control, which were significantly lower than those in the lupus (20.63 ± 4.48), ADSC (7.83 ± 2.98), and miR-20a (5.30 ± 1.88) groups. The C3 deposits in the glomeruli were decreased in the ADSC group compared to the lupus group and were the lowest in the miR-20a group (all *P* < 0.05; Figure 2(q)). The IgG deposits coincided with C3 in the glomeruli (Figure 2(q)).

### 3.3. miR-20a-ADSC Inhibits the Akt/mTOR Pathway via miR-20a

**Figure 3:**
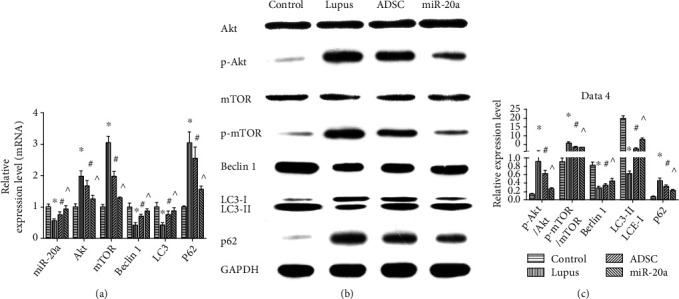
miR-20a-ADSC activates autophagy by inhibiting the miR-20a-related mTOR activation pathway in the kidney of murine LN (Akt, p-Akt, mTOR, p-mTOR, Beclin 1, LC3, and p62). (a) mRNA relative expression levels. (b) Western blot analysis of the expression of proteins in different groups. (c) The relative protein expression levels. The data are expressed as the mean ± SD. ^∗^*P* < 0.05*vs.* control group; ^#^*P* < 0.05*vs.* lupus group; ^*P* < 0.05*vs.* ADSC group. Control: normal control group; lupus: lupus group; ADSC: adipose-derived stem cell group; miR-20a: miR-20a-ADSC group.

The activated mTOR pathway has been detected in LN and has emerged as a central pathway for LN pathogenesis, and some miRNAs could regulate this pathway. Because miR-20a expression significantly decreased in the LN and miR-20a was overexpressed in miR-20a-ADSC, which also had a higher level in exosomes, we wondered whether miR-20a-ADSC has a better therapeutic effect through the paracrine activity of MSCs. RT-PCR and western blot analyses showed that the lupus group had lower miR-20a expression and higher Akt/mTOR phosphorylation levels compared to the normal group. ADSC and miR-20a-ADSC treatment resulted in the upregulated expression of miR-20a and inhibited the phosphorylation of Akt and mTOR compared with the lupus group, and the miR-20a group had a much stronger effect compared with the ADSC group (all *P* < 0.05; Figure 3).

### 3.4. miR-20a-ADSC Activates Autophagy via the mTOR Pathway

mTOR is a transcriptional regulator of autophagy and acts as an important regulator in autophagy induction. Currently, Beclin 1, LC3-II/LC3-I, and p62 have been used widely as autophagy markers. Thus, the expression levels of Beclin 1, LC3, and p62 were examined in different groups by RT-PCR and western blotting. Lower levels of Beclin 1 and LC3-II/LC3-I and higher levels of p62 were observed in the lupus group compared to the normal control group, which indicated that autophagy was inactivated in the lupus group. Compared to the lupus group, the ADSC group showed lower p62 expression and higher Beclin 1 and LC3-II/LC3-I expression (*P* < 0.05), and the miR-20a group had a much stronger effect on inducing autophagy compared with the ADSC group (*P* < 0.05, Figure 3(a)).

### 3.5. miR-20a-ADSC Reduces Podocyte Damage by Autophagy

**Figure 4:**
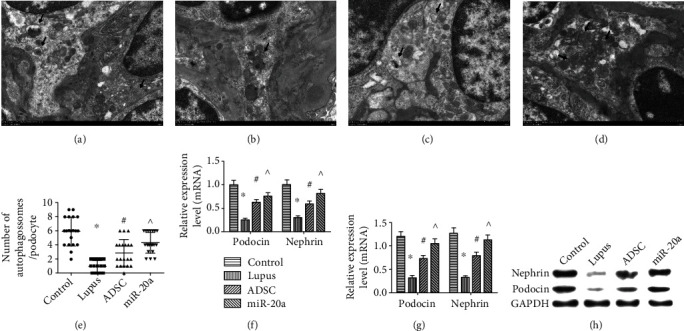
Effect of miR-20a-ADSC/ADSC treatment on the podocyte of different groups. (a–d) Transmission electron micrograph showing autophagosomes (arrows) in the podocytes from the normal control (a), lupus (b), ADSC (c), and miR-20a (d) groups (8,000x). (e) Number of autophagosomes per podocyte. (f) Nephrin and podocin mRNA relative expression levels. (g) Nephrin and podocin relative protein expression levels. (h) Western blot analysis of nephrin and podocin expression in different groups. The data are expressed as the mean ± SD. ^∗^*P* < 0.05*vs.* control group; ^#^*P* < 0.05*vs.* lupus group; ^*P* < 0.05*vs.* ADSC group. Control: normal control group; lupus: lupus group; ADSC: adipose-derived stem cell group; miR-20a: miR-20a-ADSC group.

Autophagy plays an important role in the pathogenesis of LN by affecting podocytes, T cells, B cells, dendritic cells, and phagocytes [20]. IgG and C3 deposits could harm podocytes and could be reversed by activating autophagy. We examined podocyte injury, marked by reduced expression of podocin and nephrin, in different groups. We found that podocin and nephrin expression was significantly lower in the lupus group compared with the normal control group. ADSC and miR-20a-ADSC treatment upregulated the expression of podocin and nephrin compared with the lupus group, and the miR-20a group exhibited the highest expression relative to the ADSC and lupus groups (Figures 4(f)–4(h)).

TEM imaging was used to assess the autophagic double-membrane compartments that contain lamellar structures in different groups. We found that the number of autophagosomes was significantly lower in the lupus group compared with the normal control group. The ADSC and miR-20a groups showed more autophagosomes compared with the lupus group, and the miR-20a group had the highest number of autophagosomes among groups (Figures 4(a)–4(d)). Subsequently, we selected 20 podocytes from each group for statistical analysis and found that the number of autophagosomes among different groups was significantly different, which suggests an active ongoing autophagic process (all *P* < 0.05; Figure 4(e)). Taken together, ADSC and miR-20a-ADSC reduce podocyte damage by activating autophagy (Figure 4).

## 4. Discussion

MSCs are known for their self-renewal, immunomodulation, and multilineage differentiation potential, as well as the prospect of their application in cellular and genetic therapies, and the efficacy of MSC transplantation has been verified in many diseases, such as autoimmune diseases and osteoarthritis [21, 22]. Previous studies have shown that ADSCs delay the onset of lupus symptoms, reduce proteinuria, and improve kidney histology in BWF1 and/or MRL/lpr lupus-prone strains [10, 23, 24]. Our previous study also showed that ADSC treatment can prevent the development of LN and significantly ameliorate already-established disease [10]. Accumulating evidence indicates that miR-20a regulates MSC apoptosis and proliferation, thereby enhancing their therapeutic effect [25, 26]. Kim et al. had shown that miR-20a promotes MSC proliferation by regulating the cell cycle inhibitor p21 CDKN1A, which suggests possible roles of priming methods in modulating the characteristics of MSCs by controlling the expression of miRNAs in MSCs [26]. Double overexpression of miR-19a and miR-20a in induced pluripotent stem cell-derived mesenchymal stem cells effectively preserves the left ventricular function in rats with dilated cardiomyopathy [27]. Besides, miR-20a-containing exosomes from umbilical cord MSCs alleviate liver ischemia/reperfusion injury via mTOR pathway-mediated autophagy [28]. In our study, we found that miR-20a significantly promoted ADSC proliferation and inhibited apoptosis. Besides, miR-20a-ADSC treatment had a stronger repair effect in murine LN compared with the ADSC group, as evidenced by decreased anti-dsDNA antibody, serum creatinine, and 24 h urine protein levels, nephritis scores, and C3/IgG deposits.

The expression of miR-20a, a member of the miR-17-92a cluster, is downregulated in autoimmune diseases such as SLE and multiple sclerosis and thus could be used as a biomarker for treatment response [14]. The mTOR pathway plays an important role in autoimmune diseases and is closely associated with miRNAs. miR-20a suppresses IL-17 production by inhibiting oncostatin M (OSM) and C-C motif chemokine ligand 1 (CCL1) expression and the activity of the PI3K-AKT pathway in Vogt-Koyanagi-Harada [29]. miR-20a-containing exosomes from umbilical cord MSCs alleviate liver ischemia/reperfusion injury via mTOR pathway-mediated autophagy [28]. In our study, we found that miR-20a-ADSC and ADSC treatment inhibited the phosphorylation levels of Akt and mTOR through miR-20a in the kidney of mouse lupus models.

mTOR is a transcriptional regulator of autophagy, and inactive mTOR is a key signal for autophagosome biogenesis by activating the ULK kinase complex that occurs in conjunction with the PIK3C3-BECN1-ATG14 complex [17]. Autophagy is the lysosome-dependent pathway for protein degradation and is impaired in response to metabolic stresses such as autoimmune disease, certain kidney diseases, cancer, and neurodegeneration [30]. A recent study showed that Treg cells in SLE patients exhibited increased mTOR pathway activities, whereas autophagy and the suppressor function of Treg cells were diminished [31]. However, our current understanding of the precise activity of autophagy in SLE is limited. In our study, the lupus group exhibited significantly lower autophagy expression compared with the normal control group, and ADSC/miR-20a-ADSC treatment increased autophagy activity by inhibiting the miR-20a-related Akt/mTOR pathway. Compared with the ADSC group, the miR-20a group showed higher autophagy levels, along with reduced rates of Akt/mTOR phosphorylation.

Podocytes are highly specialized epithelial cells that serve as a mechanical barrier and charge barrier and secrete soluble factors to regulate other types of glomerular cells. Autophagy plays a vital role in mouse podocyte differentiation and alleviates podocyte injury [32]. In idiopathic membranous nephropathy, sPLA2-IB and PLA2R cause podocyte injury by activating the p38MAPK/mTOR/ULK1 signaling pathway that is mediated by insufficient autophagy [33]. Liang et al. showed deficient autophagy, which was evident by LC3-II and p62 expression and the number of autophagosomes in the MPC5 cells cultured with the IgAN supernatant, along with the upregulated expression of cleaved caspase-3 and a higher apoptosis rate [34]. However, Jin et al. reported that the autophagy activity and expression pattern of autophagy-related markers in podocytes are significantly positively correlated with patients with LN types III, IV, and V–IV but negatively correlated with types II and V [30]. In the current study, there was a relatively high level of C3/IgG deposits in the glomeruli, and further research showed that podocyte injury occurs in the lupus group, and ADSC/miR-20a-ADSC treatment prevents podocyte damage. Besides, TEM imaging revealed a significant decrease in the number of autophagosomes in the lupus group compared with the control group and a significant increase after ADSC/miR-20a-ADSC transplantation, and the miR-20a group had a higher level compared to the ADSC group. The above findings indicate that the therapeutic mechanism involved in ADSC/miR-20a-ADSC treatment protects podocytes through autophagy.

The current study has several limitations. The therapeutic efficacy of MSC-derived exosomes on murine lupus nephritis has not been studied individually. Long-term survival rates and therapeutic efficacy have yet to be observed in murine lupus nephritis. Finally, protein-protein interactions of autophagy have not been studied in depth.

## 5. Conclusion

The present study demonstrates the following: (1) miR-20a-ADSC transplantation prevents the development of LN and ameliorates already-established disease. (2) miR-20a-ADSC activates autophagy by inhibiting the miR-20a-related mTOR activation pathway in the kidney of murine LN. (3) miR-20a-ADSC transplantation possibly protects podocytes by activating mTOR pathway-mediated autophagy. These findings have new, far-reaching implications for the future of MSC therapy in treating autoimmune diseases.

## Figures and Tables

**Table 1:** Sequences of oligonucleotides.

Name	Sequence	
	Forward	Reverse
GAPDH	5′-ACAATGAATACGGCTACAG-3′	5′-GTCCAGGGTTTCTTACTC-3′
mTOR	5′-CACCAGAATTGGCAGATTTG-3′	5′-CGCTTCACTTCAAACTCCAC-3′
Beclin 1	5′-GCTGTTTGGAGATCTTAGAGC-3′	5′-TTCCAGCTCCTGGATCAGCC-3′
LC3B	5′-GATAATCAGACGGCGCTTGC-3′	5′-CTCGTACACTTCGGAGATGG-3′
p62	5′-AAGTACCTGCCTGAACTC-3′	5′-ACGCGTTCTTTCAGCTTC-3′
Akt	5′-ATTCAGACTGTGGCAGATG-3′	5′-GGACACCTCCATCTCTTCAG-3′
miR-20a	5′-GTCGTATCCAGTGCGTGTCGTGGAGTCGGCAATTGCACTGGATACGAC CTACCT-3′	5′-GTAAAGTGCTTATAGTGCAG-3′
Podocin	5′-AAGGACAGATATGGGCACTGTCA-3′	5′-CCAGGAGCACCTAAGCTATGGAA-3′
Nephrin	5′-GCTCAGGGAAGACAGCAACA-3′	5′-GGGCCAGGCCTGTGGT-3′

## Data Availability

All data generated or analyzed during this study are included in this published article and Supplementary Figure 1.

## Abbreviations

SLE: Systemic lupus erythematosus
MSC: Mesenchymal stem cells
ADSC: Adipose-derived stem cells
miR-20a-ADSC: miR-20a overexpression ADSC
PBS: Phosphate-buffered solution
LN: Lupus nephritis
miRNAs: MicroRNAs
NC: Negative control
RT-PCR: Reverse transcription-polymerase chain reaction PCR
OD: Optical density
PAN: Puromycin aminonucleoside.

## Supplementary Materials

Supplementary Materials Supplementary Figure 1: original western blot. (A) Western blot of cleaved caspase-3. (B) Western blot of GAPDH. (C) Western blot of CD63. (D) Western blot of GAPDH. (E) Western blot of Akt. (F) Western blot of p-Akt. (G) Western blot of mTOR. (H) Western blot of p-mTOR. (I) Western blot of Beclin 1. (J) Western blot of LC3. (K) Western blot of p62. (L) Western blot of GAPDH. (M) Western blot of nephrin. (N) Western blot of podocin. (O) Western blot of GAPDH.
