# Supplementary material for: miR-20a Overexpression in Adipose-Derived Mesenchymal Stem Cells Promotes Therapeutic Efficacy in Murine Lupus Nephritis by Regulating Autophagy
Source: Stem Cells Int. 2021 Oct 21;2021:3746335. doi: 10.1155/2021/3746335 (PMC8553505; doi:10.1155/2021/3746335)
Supplement: Supplementary Materials — Supplementary Figure 1: original western blot. (A) Western blot of cleaved caspase-3. (B) Western blot of GAPDH. (C) Western blot of CD63. (D) Western blot of GAPDH. (E) Western blot of Akt. (F) Western blot of p-Akt. (G) Western blot of mTOR. (H) Western blot of p-mTOR. (I) Western blot of Beclin 1. (J) Western blot of LC3. (K) Western blot of p62. (L) Western blot of GAPDH. (M) Western blot of nephrin. (N) Western blot of podocin. (O) Western blot of GAPDH. [file 3746335.f1.docx]

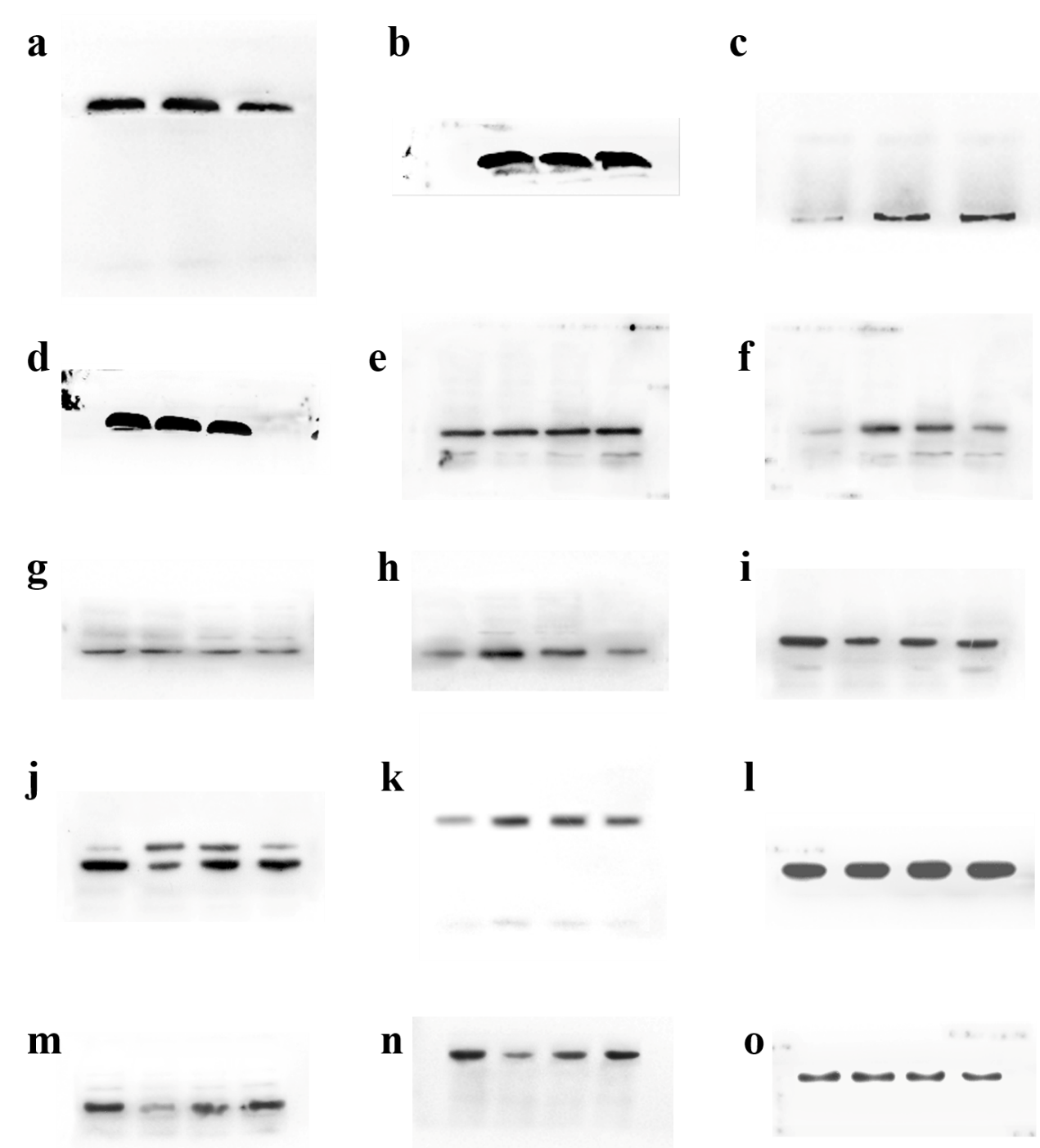


**Supplementary Figure 1**. Original Western blot. (A). Western blot of cleaved caspase-3. (B). Western blot of GAPDH. (C). Western blot of CD63. (D). Western blot of GAPDH. (E). Western blot of Akt. (F). Western blot of p-Akt. (G). Western blot of mTOR. (H). Western blot of p-mTOR. (I). Western blot of Beclin 1. (J). Western blot of LC3. (K). Western blot of p62. (L). Western blot of GAPDH. (M). Western blot of nephrin. (N). Western blot of podocin. (O). Western blot of GAPDH.
